# Transcranial Dopplers Revisited: Development of Novel Markers for Cerebral Vasospasm After Aneurysmal Subarachnoid Hemorrhage

**DOI:** 10.7759/cureus.13605

**Published:** 2021-02-28

**Authors:** Rocco Dabecco, Michael J Gigliotti, Gordon Mao, Sarah Browning, Steven Hertz, Sungyub Lew

**Affiliations:** 1 Neurosurgery, Allegheny Health Network, Pittsburgh, USA; 2 Neurological Surgery, Penn State Health Milton S. Hershey Medical Center, Hershey, USA; 3 Neurosurgery, Saint Barnabas Medical Center, Livingston, USA; 4 Vascular Surgery, Newark Beth Israel Medical Center, Newark, USA; 5 Surgery, Saint Barnabas Medical Center, Livingston, USA

**Keywords:** transcranial doppler, subarachnoid hemorrhage, aneurysm, vasospasm

## Abstract

Background

Cerebral vasospasm has been monitored by conventional angiography or transcranial Doppler (TCD). While angiography is the most accurate and reliable method for detection, TCDs are a noninvasive alternative to monitor onset and resolution of vasospasm. We aim to determine whether alternative TCD parameters rather than Lindegaard ratio lead to an improved method to diagnose and potentially prevent cerebral vasospasm.

Methods

A total of 103 consecutive patients with subarachnoid hemorrhage (SAH) were retrospectively reviewed and TCD studies were performed during the first 14 days post-bleed or longer if indicated. Multivariate logistic regression models were developed using significant univariate characteristics. Receiver operating characteristic (ROC) curves evaluated the mean middle cerebral artery (MCA), peak systolic MCA (PSV MCA), and end diastolic MCA (EDV MCA) velocities as well as ratios when compared to the ipsilateral extracranial internal carotid artery (ICA). The area under the curve was calculated to compare accuracy for symptomatic vasospasm.

Results

Thirteen patients (12.6%) were observed to develop cerebral vasospasm. Aneurysm location (p = 0.51), Hunt and Hess grade (p = 0.44), Fischer grade (p = 0.87), comorbidities, age (p = 0.67), or gender (p = 0.41) did not appear to have any effect in predicting the presence of vasospasm. ROC curves demonstrated that MCA EDV appeared to be slightly better compared to MCA velocity in predicting symptomatic vasospasm. PSV MCA/extracranial ICA and the EDV MCA/extracranial ICA ratios appeared to be an improvement to the Lindegaard ratio in the prediction of symptomatic vasospasm.

Conclusion

The utility of peak systolic and end diastolic velocities, instead of the classically referenced mean velocities and Lindegaard ratio, may improve diagnostic sensitivity of cerebral vasospasm after subarachnoid hemorrhage.

## Introduction

Cerebral vasospasm is a major cause of morbidity and death after aneurysmal subarachnoid hemorrhage (SAH) [1]. The peak frequency of cerebral vasospasm is from three to 14 days after SAH [2]. Twenty-eight percent of patients with SAH suffer clinical deterioration due to ischemic events caused by vasospasm. Of that twenty-eight percent, fifty percent of those patients either suffer long-term morbidity or they die due to delayed cerebral ischemia (DCI) [1-3]. Conventional angiography is the most accurate and reliable method for detection of vasospasm. However, it is invasive and is not without risk.

In 1982, transcranial Doppler (TCD) ultrasound was introduced by Aaslid [4] as a way to monitor the cerebral vasculature for onset and resolution of vasospasm in a noninvasive manner. There have been several studies that have attempted to correlate vasospasm with an increase in TCD velocities of the cerebral vasculature since its inception, however, the value of these studies remains controversial [3,4-7]. Despite mixed results, TCD remains the most widely used imaging modality for diagnosing cerebral vasospasm [1]. The purpose of our retrospective analysis was to evaluate alternative TCD parameters other than the classically utilized mean middle cerebral artery (MCA) velocity and mean MCA/extracranial internal carotid artery (ICA) ratio (Lindegaard ratio). The above data, when combined and analyzed along with specific clinical characteristics which may be associated with an increased risk of vasospasm, may lead to an improved method to diagnose and even prevent symptomatic vasospasm.

## Materials and methods

Patient population and demographics

A retrospective review and analysis of electronic medical records, radiographic studies, and angiographic studies was completed on 103 consecutive patients who were admitted to our institution for the treatment of spontaneous subarachnoid hemorrhage (SAH) between September 2010 and March 2015. The review of the above clinical data was performed in accordance with our institutional review board regulations (IRB Study Number 15-26, Saint Barnabas Medical Center). The diagnosis of SAH was established by CT scan or by blood or xanthochromia in the cerebrospinal fluid (CSF). Patients were excluded if they were less than 18 years of age, had poor cranial TCD windows preventing accurate analysis, and SAH caused by trauma, arteriovenous malformation, mycotic aneurysm rupture, vasculitis, or other secondary causes.

Clinical characteristics

Clinical characteristics that were analyzed included age, sex, Hunt and Hess grade, Fischer grade, time at which vasospasm occurred after SAH, day of treatment, type of treatment, and medical comorbidities (i.e., hypertension history, diabetes history, hyperlipidemia history, smoking history, and illicit drug use history). The diagnosis of symptomatic vasospasm was made as a diagnosis of exclusion, based on the development of global or focal neurological deterioration that was not explained by other causes. No retrospective diagnosis of symptomatic vasospasm was made.

Transcranial Doppler evaluation

The TCD studies were performed three days per week (Monday, Wednesday, and Friday) during the initial post-bleed course (post-bleed day 0-14). Some patients continued to undergo TCD evaluation after the typical 14-day monitoring period due to evidence of vasospasm, radiographic and/or clinical evidence of vasospasm, or due to poor clinical status and continued stay in our intensive care unit (ICU). Velocities of both the anterior and posterior circulations were measured bilaterally through the transtemporal and suboccipital windows using a 2-MHz handheld pulse-wave probe (Sonara TCD System, Natus Medical Incorporated, Middletown, WI).

Statistical analysis

Analysis was performed using IBM SPSS Statistics software, version 22 (IBM Corporation, Armonk, New York). Univariate analysis was performed on the clinical characteristics listed in Table 1. A multivariate logistic regression model was then designed using the significant univariate characteristics. Receiving operating characteristic (ROC) curves were constructed to evaluate the mean, peak systolic, and end diastolic MCA velocities and the ratios of those values when compared to the ipsilateral external internal carotid artery (ICA). The areas under the curves were then calculated to compare the accuracy of the different velocities in the detection of symptomatic vasospasm.

## Results

**Figure 1:**
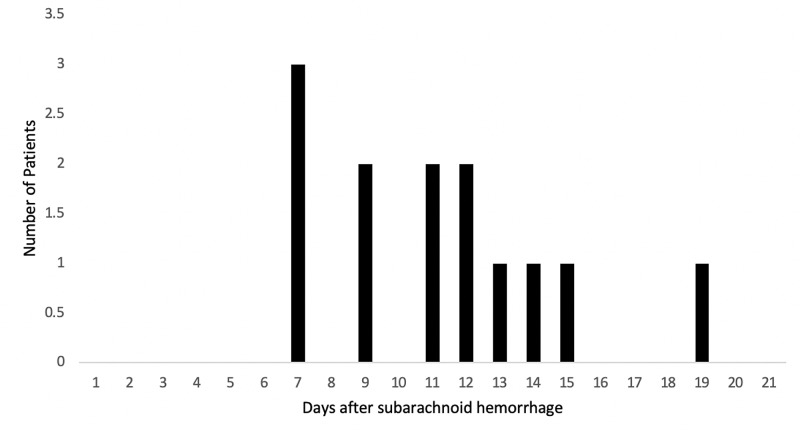
Distribution of patients developing symptomatic vasospasm according to day number post-subarachnoid hemorrhage.

**Figure 2:**
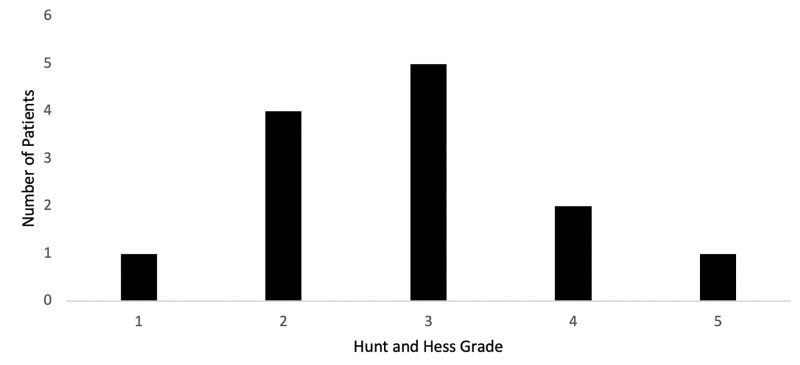
Distribution of patients developing vasospasm according to Hunt and Hess Grade.

A retrospective review of 103 consecutive patients presenting to our institution with SAH was completed. Of these patients, 76 were female (74%) and 27 were male (26%) with an age range of 25 to 89 (mean = 57). Vasospasm was observed in 13 patients (12.6%) compared to 90 patients (87.4%) without cerebral vasospasm. The most common post-bleed day in which symptomatic, cerebral vasospasm was detected was day 7 (Figure 1) and the most common Hunt and Hess grade at which vasospasm occurred was grade 3 (Figure 2).

**Figure 3:**
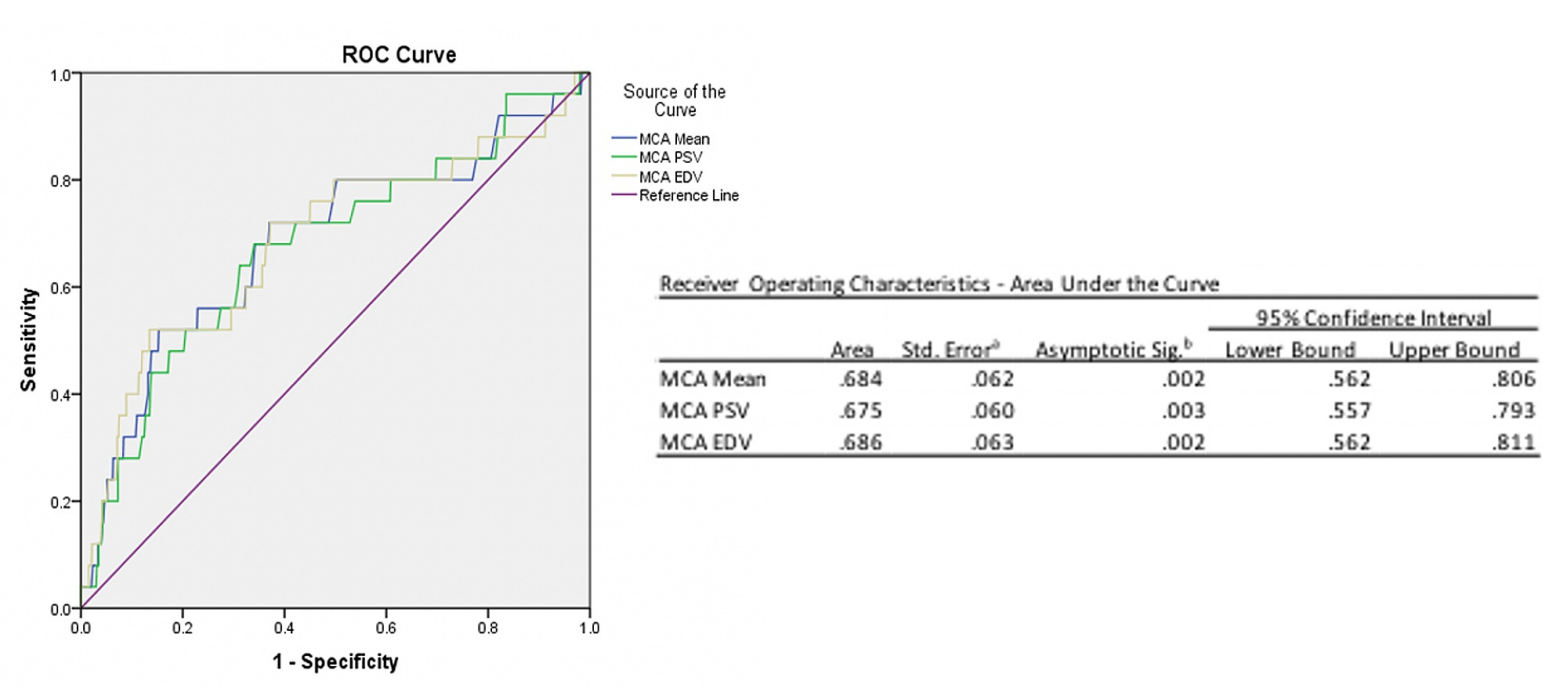
Receiver operating characteristic (ROC) curve demonstrating slight MCA EDV improvement (0.686, 95% CI: 0.562 – 0.811) to MCA mean velocity (0.684; 95% CI: 0.562 – 0.806) to predict symptomatic velocity. MCA: Middle cerebral artery; EDV: End diastolic velocity.

**Figure 4:**
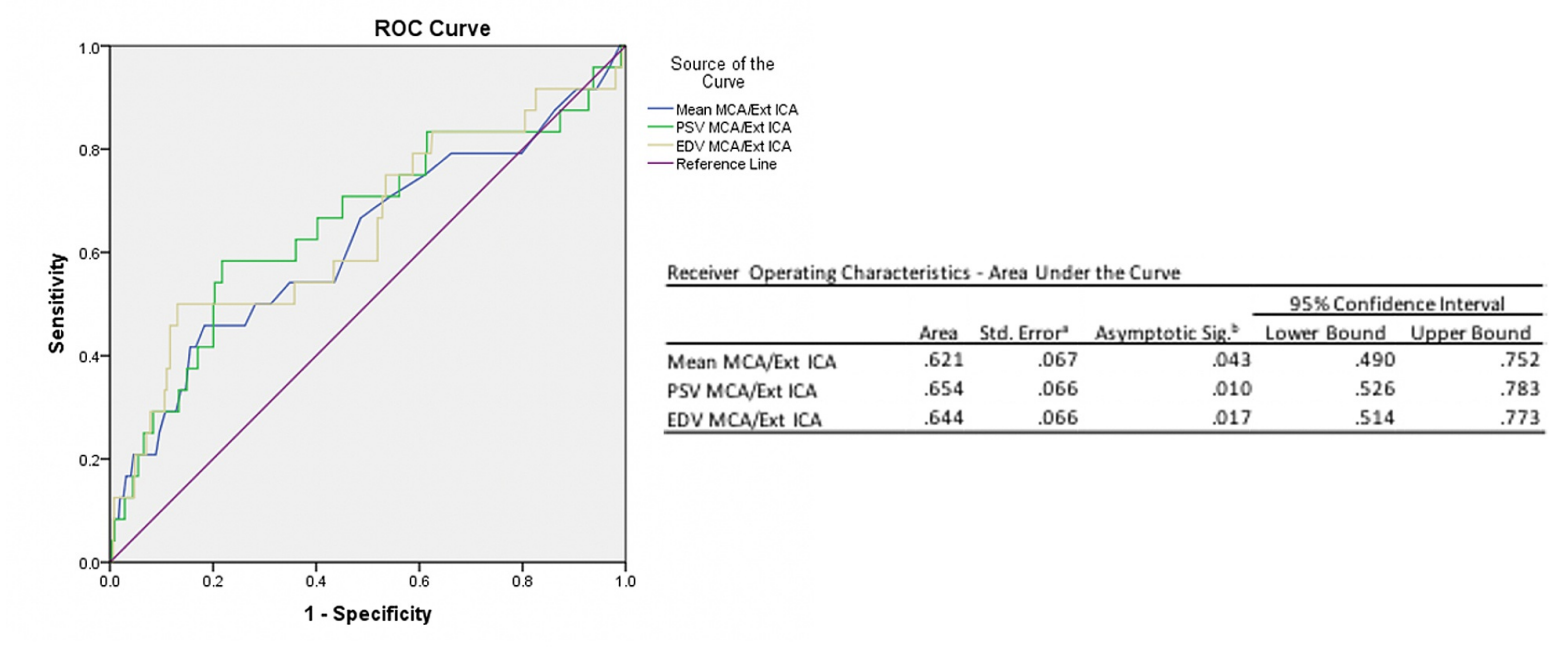
Receiver operating characteristic (ROC) curve demonstrating PSV MCA/extracranial ICA (0.654; 95% CI: 0.526 – 0.783) and the EDV MCA/extracranial ICA (0.644; 95% CI: 0.514 – 0.773) ratios appear to be better at predicting symptomatic vasospasm compared to the classically utilized Mean MCA/extracranial ICA ratio (0.621; 95% CI: 0.490 – 0.752). MCA: Middle cerebral artery; ICA: Internal carotid artery; PSV: Peak systolic velocity.

Anterior communicating artery (ACOMM) aneurysms and hypertension represented the most common aneurysm and comorbidity associated with vasospasm, respectively. There did not appear to be a statistically significant increased risk of detection of cerebral vasospasm based on location of the aneurysm (p = 0.51), Hunt and Hess grade (p = 0.44), Fischer grade (p = 0.87), comorbidities, age (p = 0.67), or gender (p = 0.41). A complete, univariate analysis of patient demographic information is in Table 1. Multivariate analysis yielded two ROC curves (Figures 3-4).

**Table 1:** Patient demographics presenting with subarachnoid hemorrhage (with or without cerebral vasospasm). EDV: End diastolic velocity; ACOMM: Anterior communicating artery; ICA: Internal carotid artery; MCA: Middle cerebral artery.

	Overall	No vasospasm	Vasospasm	p-value
Age	57 (14.1%)	57 (14.4%)	56 (12.3%)	0.669
Gender				
Male	27 (0.2%)	25 (0.3%)	2 (0.2%)	0.405
Female	76 (0.7%)	65 (0.7%)	11 (0.8%)	
Comorbidity				
Hypertension	61 (0.6%)	54 (0.6%)	7 (0.5%)	0.981
Diabetes	20 (0.2%)	17 (0.2%)	3 (0.2%)	0.863
Hyperlipidemia	32 (0.3%)	28 (0.3%)	4 (0.3%)	0.969
Drug Use	5 (0%)	4 (0%)	1 (0.1%)	0.827
Smoker	42 (0.4%)	37 (0.4%)	5 (0.4%)	0.982
Family History	2 (0%)	2 (0%)	0 (0%)	0.861
EDV	61 (0.6%)	50 (0.5%)	11 (0.8%)	0.082
Mortality	20 (0.2%)	18 (0.2%)	2 (0.2%)	0.651
Hunt & Hess grade				0.442
1	22 (0.2%)	21 (0.2%)	1 (0.1%)	
2	28 (0.3%)	24 (0.3%)	4 (0.3%)	
3	26 (0.3%)	21 (0.2%)	5 (0.4%)	
4	10 (0.1%)	8 (0.1%)	2 (0.2%)	
5	18 (0.2%)	17 (0.2%)	1 (0.1%)	
Fischer grade				0.873
1	4 (0%)	4 (0%)	0 (0%)	
2	17 (0.2%)	15 (0.2%)	2 (0.2%)	
3	25 (0.2%)	22 (0.2%)	3 (0.2%)	
4	57 (0.6%)	49 (0.5%)	8 (0.6%)	
Location				0.514
ACOMM	22 (0.2%)	3 (0.2%)	25 (0.2%)	
ICA	8 (0.1%)	2 (0.2%)	10 (0.1%)	
MCA	12 (0.1%)	3 (0.2%)	15 (0.1%)	
Posterior circulation	18 (0.2%)	3 (0.2%)	21 (0.2%)	
Other	36 (0.4%)	2 (0.2%)	38 (0.3%)	

Figure 3 demonstrated that MCA EDV (0.686; 95% CI: 0.562 - 0.811) was slightly improved when compared to MCA mean velocity (0.684; 95% CI: 0.562 - 0.806) in the prediction of symptomatic vasospasm. Alternatively, Figure 4 demonstrated that PSV MCA/extracranial ICA (0.654; 95% CI: 0.526 - 0.783) and the EDV MCA/extracranial ICA (0.644; 95% CI: 0.514 - 0.773) ratio showed an improvement to predicting symptomatic vasospasm compared to the Lindegaard ratio (0.621; 95% CI: 0.490 - 0.752).

## Discussion

Symptomatic vasospasm remains the major cause of morbidity and mortality in patients with SAH. The goal of TCD monitoring is to identify patients at risk for vasospasm, enabling prompt and effective treatment of these patients. Suarez et al. were able to demonstrate that TCD velocities can be utilized to identify vasospasm at a mean of 24 hours before the presence of symptomatic vasospasm, showing that there is predictive power in this tool as well [8]. The studies typically referenced in the literature use the mean velocities for analyzing the risk of vasospasm. In our study, we aimed to evaluate the accuracy of non-traditional intracranial blood flow parameters against the historical benchmarks of the mean MCA velocity and Lindegaard ratio in predicting significant vasospasm after aneurysm rupture.

Our multivariate analysis eventually identified the MCA EDV and MCA PSV/extracranial ICA as new candidates for predictors of DCI and symptomatic vasospasm. Using the ROC, we saw that the accuracy (ROC area underneath the curve) was improved for MCA EDV (0.686) compared to the MCA mean (0.684) and the MCA PSV/extracranial ICA (0.654) as well as MCA EDV/extracranial ICA (0.644) versus the mean MCA/extracranial ICA (0.621). However, the 95% CI demonstrated overlap between these new candidate measures and the traditional mean MCA and Lindegaard ratio indicating a trend toward significance. Future analysis to determine whether these alternative means to predicting intracranial vasospasm are in fact an improvement over traditional measures should include more data points as more patients undergo TCD monitoring for SAH.

Another area that we would like to concentrate on with further analysis is examining the percentage change in velocities both from a baseline value (the initial TCD study) and on a day-to-day basis. We would like to take that possible predictive value demonstrated in prior studies one step further, ultimately analyzing possible threshold values or cut-off points that determine the risk of vasospasm for patients with SAH. This analysis may be similar to Malhotra et al., who demonstrated that an absolute increase by >50 cm/second over the course of 48 hours was significant in predicting vasospasm [9]. However, percent changes from baseline and day-to-day percentage changes were poor predictors.

## Conclusions

Reviewing and analyzing five years of clinical, angiographic, radiographic, and TCD data has shown us that utilizing the peak systolic and end diastolic velocities, instead of the classically referenced mean velocities and Lindegaard ratio, may improve the diagnosis of cerebral vasospasm after subarachnoid hemorrhage. As we continue to add new patients in our database, we anticipate that the power of our study will continue to increase to better support the diagnostic superiority of our current study variables over the traditional measures. Moreover, future directions should be dedicated to evaluating the possibility of accurately predicting vasospasm, prior to the onset of clinical symptoms, utilizing the change in velocity from the patient’s baseline TCD as a comparison.
